# ApoE Genotype-Dependent Response to Antioxidant and Exercise Interventions on Brain Function

**DOI:** 10.3390/antiox9060553

**Published:** 2020-06-25

**Authors:** Kiran Chaudhari, Jessica M. Wong, Philip H. Vann, Tori Como, Sid E. O’Bryant, Nathalie Sumien

**Affiliations:** 1 Department of Pharmacology and Neuroscience, University of North Texas Health Science Center, Fort Worth, TX 76107, USA; kchaudha@live.unthsc.edu (K.C.); Jessica.Wong@unthsc.edu (J.M.W.); Philip.Vann@unthsc.edu (P.H.V.); 2 Institute for Translational Research, Department of Pharmacology and Neuroscience, University of North Texas Health Science Center, Fort Worth, TX 76107, USA; tori.como@unthsc.edu (T.C.); sid.Obryant@unthsc.edu (S.E.O.)

**Keywords:** ApoE, exercise, antioxidants, oxidative stress, cognition, motor, vitamin E, vitamin C, aging, Alzheimer’s disease

## Abstract

This study determined whether antioxidant supplementation is a viable complement to exercise regimens in improving cognitive and motor performance in a mouse model of Alzheimer’s disease risk. Starting at 12 months of age, separate groups of male and female mice expressing human Apolipoprotein E3 (GFAP-ApoE3) or E4 (GFAP-ApoE4) were fed either a control diet or a diet supplemented with vitamins E and C. The mice were further separated into a sedentary group or a group that followed a daily exercise regimen. After 8 weeks on the treatments, the mice were administered a battery of functional tests including tests to measure reflex and motor, cognitive, and affective function while remaining on their treatment. Subsequently, plasma inflammatory markers and catalase activity in brain regions were measured. Overall, the GFAP-ApoE4 mice exhibited poorer motor function and spatial learning and memory. The treatments improved balance, learning, and cognitive flexibility in the GFAP-ApoE3 mice and overall the GFAP-ApoE4 mice were not responsive. The addition of antioxidants to supplement a training regimen only provided further benefits to the active avoidance task, and there was no antagonistic interaction between the two interventions. These outcomes are indicative that there is a window of opportunity for treatment and that genotype plays an important role in response to interventions.

## 1. Introduction

Apolipoprotein E4 (ApoE4) is the most prevalent genetic risk factor for late-onset Alzheimer’s disease (AD) [1]. In mice expressing human ApoE4, the cognitive deficits can be measured in terms of impairments in spatial learning and memory [2] and working memory [3]. Apart from cognitive declines, other behavioral effects are associated with AD and preclinical AD, such as increased anxiety [4], motor function disability, and the inability to learn new motor skills [5,6]. Interestingly, the presence of the ε4 allele was also associated with a two-fold increase in the rate of global motor function decline when compared with non-carriers with comparable age, sex, and education [7].

Oxidative stress has been associated with cognitive and motor declines and has been suggested as a major contributor to AD pathology. Oxidative stress has also been involved in vascular cognitive impairment, a risk for dementia [8]. The brains of AD patients are more vulnerable to oxidative stress, as evidenced in animal models and humans [9]. Furthermore, ApoE4 is associated with the aggravation of AD pathophysiology via increased oxidative stress [10]. Therefore, AD symptomology, especially in the presence of the ApoE4 allele, should be responsive to therapies reducing oxidative stress. Vitamin E is an example of such a therapeutic known to reduce oxidative stress and is able to improve cognitive function in AD patients either alone or in combination [11].

Another well-marketed healthy lifestyle modification is exercise. Physical activity has been associated with a reduced risk of AD [12], delayed AD onset [13], and improved AD symptoms [14], as well as improved vascular cognitive impairments [8]. Furthermore, exercise was even more beneficial in ApoE4 carriers than non-carriers [15,16]. Exercise lowered oxidative stress [17] and improved cognition [18], but also reduced anxiety in the elderly [19] and in rats [20]. Furthermore, it improved motor function in a cognitively impaired geriatric population [21,22], and motor training dramatically reduced injurious falls among AD patients [23,24]. Oxidative stress has also been implicated in the development of neuromuscular disorders (NMDs), and exercise in an intensity- and duration-dependent manner can alter NMDs, as reviewed by Siciliano et al. [25].

Both lines of therapy improved behavioral outcomes associated with AD, and seemed to at least partially involve oxidative stress as part of their mechanism of action. Therefore, it can be hypothesized that combining antioxidants with exercise training will lead to an additive beneficial effect, reducing impairments. While some reports have determined that there can be such a positive interaction [26,27,28], other studies have found a negative relationship, in which the presence of antioxidants negated the beneficial effects of exercise [29]. How such combinations affect behavioral measures have not been fully explored, and the influence of genotype and sex remain to be evaluated.

The goals of the current study were to determine (1) cognitive, motor, and anxiety phenotypes of middle-aged GFAP-ApoE3 and E4 male and female mice; (2) whether antioxidant intake or exercise training leads to functional improvements; (3) whether the combination of antioxidant and exercise yields an additive beneficial effect; (4) the involvement of oxidative stress and inflammation in behavioral outcomes. Our hypothesis was that moderate exercise and antioxidants would lead to additional beneficial effects when compared to each intervention alone, which would be exacerbated in the ApoE4 genotype. The outcomes are important in deciding the need for antioxidant supplementation in exercising individuals, and as a guiding parameter in genotype-based experiments.

## 2. Materials and Methods

### 2.1. Animals

All animal use protocols were approved by the Institutional Animal Care and Use Committee at UNT HSC (Protocol #: 2009/10-50-A04, approved 09/1/2010). Groups of male and female GFAP-ApoE3 (B6.Cg-Tg(GFAP-*APOE*3)37Hol *APOE*tm1Unc/J) and GFAP-ApoE4 (B6.Cg-Tg(GFAP-*APOE*4)1Hol *APOE*tm1Unc/J) mice were obtained from The Jackson Laboratory (Bar Harbor, ME, USA) (catalog numbers 004633 and 004631; we started with a total of 187 mice, and recorded 14 and 10 deaths for the ApoE3 and ApoE4 respectively) at 2 months. These mice express the human apolipoprotein E3 or E4 under the control of the glial fibrillary acidic protein (GFAP) promoter and do not express endogenous ApoE. The mice were group housed in standard housing conditions and received ad libitum access to food and water at an ambient temperature, under a 12-h light/dark cycle starting at 06:00.

### 2.2. Treatment

When the mice were 12 months old, they were randomly allotted to: (1) sedentary + control diet (Sed-Con), (2) sedentary + control diet supplemented with vitamins E and C (Sed-EC), (3) exercise + control diet (Ex-Con), (4) exercise + control diet supplemented with vitamins E and C (Ex-EC). The diets were obtained from TestDiet (St Louis, MO, USA). The control diet (LabDiet^®^ R&M 5LG6 4F, #5S84) was modified by adding ascorbic acid (1.65 mg/g diet) and α-tocopheryl acetate (1.12 IU/g diet) (#5SH0). Using treadmills, the mice were progressively introduced to a moderate exercise regimen (AccuPacer Treadmill, Omnitech Electronics, Inc., Columbus, OH, USA). The training was increased in time and speed to reach a maximal exercise of 1 h over 12 days (6, 8, 10, and 12 m/min for 5 min each, and then at 14 m/min for 40 min). Compliance was achieved by a transient 0.29 mA electric foot shock to the feet. The number of shocks were tallied, and the paired control mice received the same number of shocks/training day.

### 2.3. Food Intake and Body Weights

The mice were weighed weekly and food intake (average of five consecutive days) was recorded the week before the start of behavioral testing.

### 2.4. Functional Testing

For 16 weeks, the mice were on their respective treatments, including 8 weeks prior to the behavioral assessments. The behavioral tasks were described in details previously [30,31].

#### 2.4.1. Elevated Plus Maze

The position of a mouse in the maze (two open arms and two closed arms, 3ft above the floor) was determined by a tracking system (Any-maze, Stoelting Co., Wood Dale, IL, USA). Under dim light (60 W) and for 5 min, each mouse was left to explore. Time in open arms was used to measure anxiety levels.

#### 2.4.2. Spontaneous Activity

Each mouse was left to explore an acrylic test chamber (40.5 cm × 40.5 cm × 30.5 cm) under dim light and with background noise (80 dB) for 16 min. Their activity was recorded using a Digiscan apparatus (Omnitech Electronics Inc., Columbus, OH, USA, model RXYZCM-16). Their horizontal, vertical, and spatial movements were detected by the photocells and processed by a software program.

#### 2.4.3. Coordinated Running

A motor-driven cylinder rotating at increasing speed was used to measure motor learning and running performance (Rotorod, Omnitech Electronics Inc., Columbus, OH, USA, Model # AIO411RRT525M). The mice were given two training sessions per day (four trials/session with a 10 min inter-trial intervals (ITI)), which continued until improvements failed over three consecutive sessions. The average of latency to fall for the four trials in each session was used for motor learning and for the final session when stable performance was achieved.

#### 2.4.4. Reflexive Musculoskeletal Responses

Walk initiation: record the latency to move one body length immediately after being placed on a smooth surface. Alley turn: record the latency to make a full turn in a dead-end alley. Negative geotaxis: record the latency to turn 180 ° in either direction when placed facing downward on a 45 ° tilted grid. Wire suspension: record the latency to grasp wire with hindpaws after being suspended from wire by front paws and the latency to fall (two trials/session). The mice received four sessions (one/day) and latencies were averaged across the four sessions. A maximum of 60 s for each test was used.

#### 2.4.5. Bridge Walking

Each mouse was placed on one of four bridges (large square, small square, large round, small round) and latency to fall (maximum of 60 s) was recorded. Each bridge was presented three times and latency to fall was averaged for each bridge and across bridges.

#### 2.4.6. Morris Water Maze (MWM)

Spatial learning and memory were measured using a modified Morris water maze test. Prior to any testing, the mice received pre-training using a covered straight alley to teach the mice to swim and climb onto a platform. Following this, the mice were given a maximum of 90 s to find a hidden platform from different starting positions (one session/day, five trials/session with a 2 min ITI). Their performance was tracked via Any-maze software (Stoelting Co., Wood Dale, IL, USA). Path length and swim speed were used as measures of performance.

#### 2.4.7. Discriminated Avoidance

Using a T-maze apparatus set on a grid floor set to deliver 0.69 mA scrambled shock, we tested the mice for learning and cognitive flexibility using a discriminated avoidance task. During the information trial (first one), a shock is administered when the mouse enters their preference arm (first one) and allowed to escape the shock by running to the opposite arm (correct arm). Thereafter, the initiation of shock was 5 s upon start door opening or upon entry into an incorrect arm until the mouse entered the correct arm or a maximum of 60 s. After 10 s in the correct arm, the mouse was placed in a holding cage for 1 min (ITI). Training continued until a criterion of correct avoidance was reached (choosing the correct arm in under 5 s in four out of five trials, with the last two being correct avoidances). The reversal sessions followed the same training and switching of the correct arm. Learning ability was considered inversely proportional to the number of trials required to reach the avoidance criterion.

### 2.5. Biochemical Measurements

After behavioral testing, blood was collected via tail bleed prior to euthanization (by cervical dislocation). Brains were harvested and dissected into brain regions that were saved at −80 °C (cerebral cortex, striatum, cerebellum, and hippocampus).

#### 2.5.1. Catalase Activity

The activity of the antioxidative enzyme, catalase, was evaluated using the Catalase Assay Kit from Cayman Chemical (Ann Arbor, MI, USA, catalog number 707002). Methanol was reacted with catalase in the presence of for the optimal concentration of H_2_O_2_. The production of the resulting formaldehyde was measured colorimetrically with Purpald (4-amino-3-hydrazino- 5-mercapto-1,2,4-triazole) at 540 nm.

#### 2.5.2. Inflammatory Markers

Plasma levels for IL-10, IL-6, and TNFα were measured using an MSD Inflammatory Panel Kit from Meso Scale Diagnostics, LLC, Rockville, MD, USA) (10-plex Proinflammatory Panel 1 (mouse) kit (cat # C4048-1) and analyzed by MSD DISCOVERY WORKBENCH^®^ analysis software (Meso Scale Diagnostics, Rockville, MD, USA). While the panel measures more than these four markers, the others were out of range based on the manual and literature searches. Therefore, we did not include them in our analyses.

### 2.6. Statistical Analysis

The functional performance of the mice on the behavioral tests, as well as the biochemical measurements, were assessed using three-way analyses of variance (ANOVA) with sex, genotype, and treatment as between-group factors (or genotype and treatment for catalase measurements due to low *n*). Planned individual comparisons between different sexes, genotypes, and treatments were performed using single degree-of-freedom *F*-tests involving the error term from the overall ANOVA. Some behavioral performances and catalase activity were also considered in four-way analyses with session or brain region as the repeated measure. The software used for the analyses was Systat 13 (San Jose, CA, USA) and *p* was set< 0.05.

## 3. Results

### 3.1. Body Weight and Food Intake

**Figure 1:**
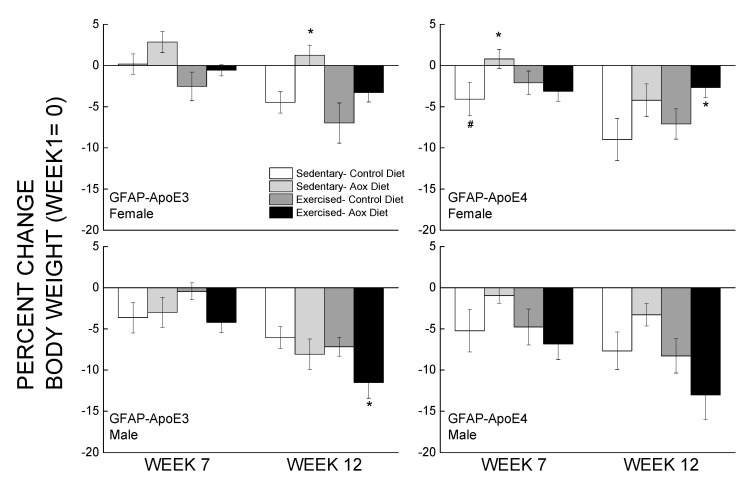
Minimal effects of exercise and/or antioxidant regimen over time on body weights of middle-aged GFAP-APOE3 and GFAP-APOE4 male and female mice (mice expressing the human apolipoprotein (Apo) E3 or E4 under glial fibrillary acidic protein (GFAP) promoter control). Each value represents mean ± SEM, *n* = 5–16 for body weights, and *n* = 3–7 for food intake. * *p* < 0.05 vs. sex- and strain-matched Sedentary-Control (Sed-Con) groups; # *p* < 0.05 comparing sex-matched Sed-Con GFAP-ApoE3 and GFAP-ApoE4.

Percent changes in body weight from the week before behavioral testing started and at the end of the study are presented in Figure 1. Overall, male mice weighed more than female mice, and within the female groups, GFAP-ApoE4 mice weighed less than the GFAP-ApoE3 mice (not shown). These observations were supported by main effects of strain, sex, and an interaction between sex and strain (all *p* < 0.02). By week 7, most mice lost 3–7% of their body weight, and the only significant differences were between E3 and E4 Sed-Con females, and between Sed-Con and Sed-Aox in the GFAP-ApoE4 females (all *p* < 0.05). By week 12, the loss was more pronounced (up to 13%), and the only significant differences were Ex-Aox GFAP-ApoE4 females losing less weight than Sed-Con (*p* < 0.05), while in males, the Ex-Aox groups lost the most weight (*p* < 0.05 only for GFAP-ApoE3). Food intake was not significantly affected by sex, strain or treatment (all *p* > 0.2) (not shown).

### 3.2. Behavioral Measurements

#### 3.2.1. Elevated Plus Maze

The performance of the mice in this test measuring affective function was analyzed and is presented in Figure S1. The GFAP-ApoE3 and GFAP-ApoE4 mice spent the same amount of time in the open arms, however, males spent more time in the open arm than females, supported by a main effect of sex (*p* < 0.001) and no effect of strain (*p* = 0.366). Treatments did not affect performance (all *p* > 0.185), with the exception of Sed-Aox group spending more time than the Sed-Con group in the open arms (*p* = 0.046). Overall, the GFAP-ApoE3 mice were more active than the GFAP-ApoE4 mice, and some treatments affected the activity of females (all *p* < 0.003). These observations were supported by the significant main effects of strain and treatment and a sex × strain × treatment interaction. (all *p* < 0.01).

#### 3.2.2. Spontaneous Activity

Horizontal distance and rearing activity were analyzed (Figure S2). GFAP-ApoE4 mice travelled 40% shorter distances than the GFAP-ApoE3 mice, and GFAP-ApoE3 females traveled 18% more than their male counterparts. Treatment had no effect on distance traveled. An ANOVA yielded a main effect of strain (*p* < 0.001) and no effect for treatment, sex or any interactions between the factors (all *p* > 0.300). GFAP-ApoE4 mice also spent 20% less time rearing than the GFAP-ApoE3, supported by a main effect of strain (*p* = 0.001). Most treatments had no effect on the rearing activity of the mice with the exception of Sed-Aox, which increased it (*p* = 0.046).

#### 3.2.3. Coordinated Running Performance

**Figure 2:**
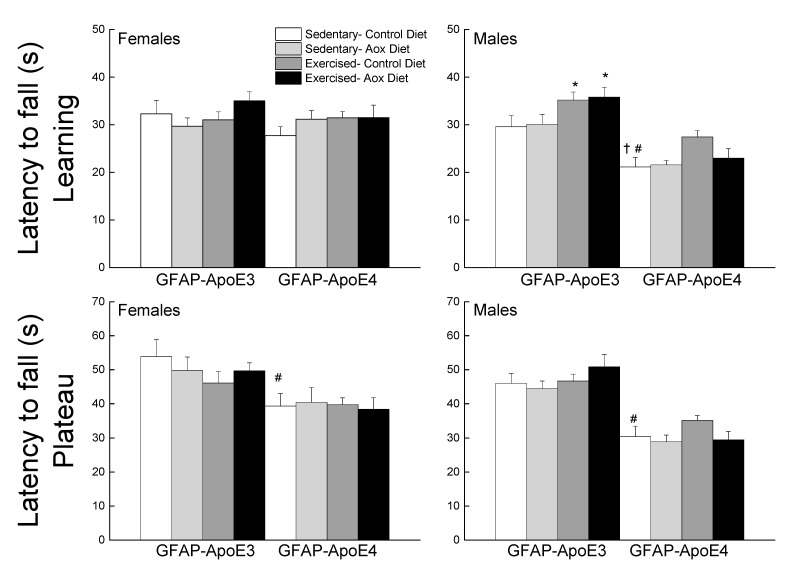
Exercise improved learning performance in male GFAP-ApoE3 and GFAP-ApoE4 mice, but not plateau performance. Each value represents mean ± SEM, *n* = 8–16. * *p* < 0.05 vs. sex- and strain-matched Sed-Con groups; # *p* < 0.05 comparing sex-matched Sed-Con GFAP-ApoE3 and GFAP-ApoE4 mice; † *p* < 0.05 comparing strain-matched Sed-Con males and females.

The data were averaged as learning (sessions 1–4) and plateau (sessions 5–7) performance (Figure 2). During the learning phase, GFAP-ApoE4 mice fell faster from the rod than the GFAP-ApoE3 mice, especially males, supported by the main effects of sex, strain and a sex x strain interaction (all *p* < 0.003). A main effect of treatment (*p* = 0.014) was due to Ex-Con and Ex-Aox GFAP-ApoE3 mice exhibiting better performance in males (all *p* < 0.04). The Ex-Con GFAP-ApoE4 group also seemed to have a better performance (*p* = 0.051) which was not seen in the Ex-Aox group. During the plateau phase, there was only an effect of strain, in which GFAP-ApoE4 mice had a poorer performance than the GFAP-ApoE3 mice (*p* < 0.001).

#### 3.2.4. Reflexive Musculoskeletal Responses

Performance was averaged over four sessions and is presented in Figure S3. GFAP-ApoE4 mice took longer latencies than the GFAP-ApoE3 mice to initiate walking, supported by a main effect of strain (*p* = 0.019). There was no effect of treatment, sex, or interactions between any of the factors (all *p* > 0.086). Alley-turning was affected differentially by the treatments depending on sex and genotype (sex × strain × treatment interaction, *p* = 0.004). GFAP-ApoE4 females took longer latencies than the GFAP-ApoE3 females, while there was no difference in males. In GFAP-ApoE4 females, Sed-Aox took longer to turn, while in GFAP-ApoE3, the Ex-Aox males had the shortest latency. For negative geotaxis, males took longer latencies to turn, as well as GFAP-ApoE4 mice. The Sed-Aox mice had shorter latencies in the GFAP-ApoE3 groups (strain × treatment, *p* = 0.058). Latency to tread was affected by strain (*p* = 0.01), due mainly to treatments affecting each genotype differently (higher in GFAP-APoE4 males vs. lower latencies in GFAP-ApoE3 mice). There was no effect of treatment or interactions between any of the factors (all *p* > 0.10).

#### 3.2.5. Bridge Walking

**Figure 3:**
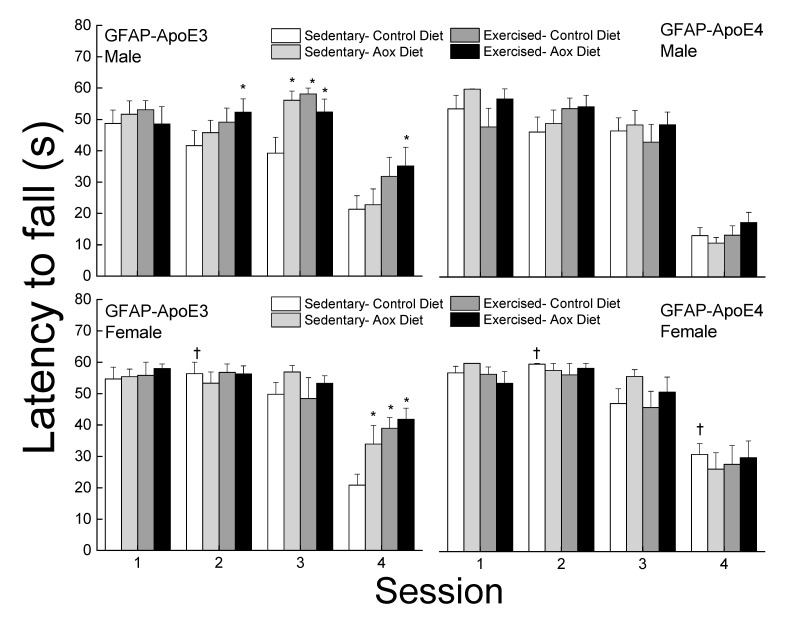
Exercise and antioxidants improved balance in male and female GFAP-ApoE3 mice but not in GFAP-ApoE4 mice. Each value represents mean ± SEM, *n* = 8–16. * *p* < 0.05 vs. sex- and strain-matched Sed-Con groups; † *p* < 0.05 comparing strain-matched Sed-Con males and females.

The latency to fall was analyzed for each bridge and is presented in Figure 3. On the easiest bridge (session 1), there was no effect of strain, sex, or treatment on the latency to fall (all *p* > 0.13). In session 2, females performed better than males leading to a main effect of strain. While there was no main effect of treatment, exercised groups had higher latencies in males regardless of strain but it only reached significance for the Ex-Aox GFAP-ApoE3 mice (*p* = 0.029). In session 3, a main effect of treatment was obtained (*p* = 0.03) due to all treatments in male GFAP-ApoE3 mice. In session 4, treatments, especially exercise, were associated with increased latencies in the GFAP-ApoE3 but not GFAP-ApoE4 groups. GFAP-ApoE4 females performed better than their male counterparts, driving the main effects of sex and strain (all *p* < 0.03).

#### 3.2.6. Morris Water Maze

**Figure 4:**
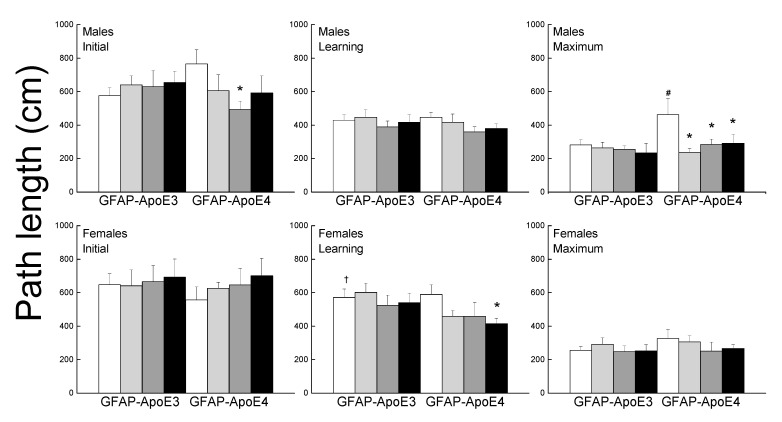
Exercise and antioxidants were associated with mild improvement in swim maze performance in male GFAP-ApoE4 mice but not in GFAP-ApoE3 mice. Each value represents mean ± SEM, *n* = 5–16. * *p* < 0.05 vs. sex- and strain-matched Sed-Con groups; # *p* < 0.05 comparing sex-matched Sed-Con GFAP-ApoE3 and GFAP-ApoE4 mice; † *p* < 0.05 comparing strain-matched Sed-Con males and females.

The performance of the mice was separated into initial, learning phase, and maximum performances (Figure 4). During the initial session, there was no effect of strain or treatment for females, however, in the males, all treatment improved performance but it only reached significance for Ex-Con (*p* = 0.044). During the learning phase, female mice had higher path lengths than the males (main effect of sex; *p* < 0.001). Treated GFAP-ApoE4 females had lower latencies than the sex- and strain-matched Sed-Con group (*p* = 0.064), however, it only reached significance with the Ex-Aox mice. Maximum performance was impaired in the GFAP-ApoE4 males, and was reversed by all treatments. However, an ANOVA did not reach significance for strain (*p* = 0.098) or strain x treatment (*p* = 0.096). Females swam slower than males, GFAP-ApoE4 mice seemed slightly faster than GFAP-ApoE3 mice, and none of the treatments affected the swimming speed (not shown). ANOVA yielded a main effect of sex (all *p* < 0.007), but the effect of strain did not reach significance (*p* = 0.057).

#### 3.2.7. Discriminated Avoidance Test

**Figure 5:**
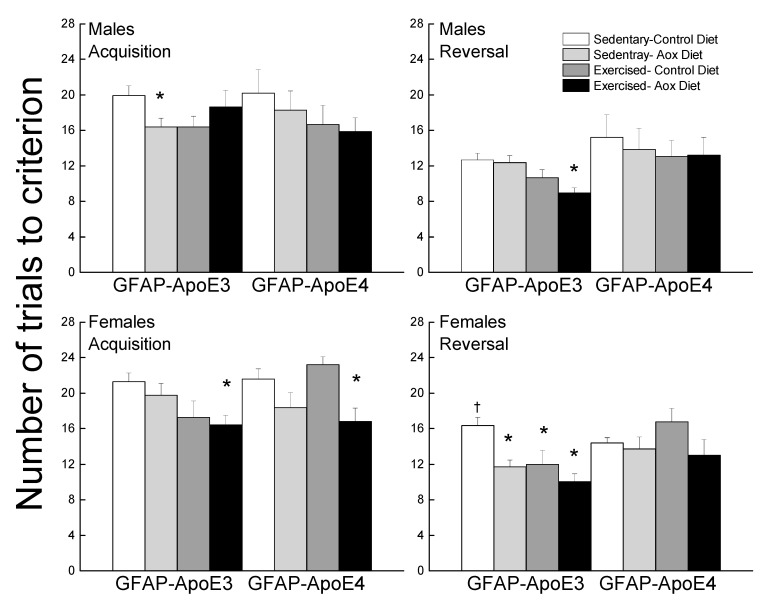
Exercise and antioxidants improved learning and cognitive flexibility in male and female GFAP-ApoE3 mice but not in GFAP-ApoE4 mice. Each value represents mean ± SEM, *n* = 8–16. * *p* < 0.05 vs. sex- and strain-matched Sed-Con groups; † *p* < 0.05 comparing strain-matched Sed-Con males and females.

Performance during acquisition and reversal is presented in Figure 5. During acquisition, most treatments improved performance (main effect of treatment; *p* = 0.005). Sed-Aox (*p* = 0.035) and Ex-Con (*p* = 0.052) groups took fewer trials to reach criterion in the GFAP-ApoE3 males and there was no significant effect in the GFAP-ApoE4 mice. In females, Ex-Con (*p* = 0.058) and Ex-Aox (*p* = 0.019) performed better than the Sed-Con group in the GFAP-ApoE3 group, while only Ex-Aox (*p* = 0.018) took fewer trials than the controls in the GFAP-ApoE4 group.

During reversal, treatments improved performance (main effect of treatment; *p* = 0.007), mainly in the GFAP-ApoE3 mice, but this observation was not supported by an interaction between strain and treatment (*p* = 0.23). There was no significant effect of any of the treatments in the GFAP-ApoE4 mice. In the GFAP-ApoE3 group, all treated mice took fewer trials compared to Sed-Con in females and Ex-Aox was the only significantly different group in males (*p* = 0.029).

### 3.3. Biochemical Measurements

#### 3.3.1. Catalase

Catalase activity was measured in the cerebral cortex, hippocampus, cerebellum, and midbrain and is presented in Figure S4 (due to low *n*, sexes were combined). Overall, the activity was ranked as follows: hippocampus > cerebellum, midbrain > cortex, supported by a main effect of region (*p* < 0.001). There was an overall effect of treatment (*p* = 0.003) which was not region-dependent (all *p* > 0.96), but no effect of genotype (all *p* > 0.35). The effect of treatment was due mostly to effects of Ex-Aox in all regions (not significant for cerebellum).

#### 3.3.2. Inflammatory Markers

**Figure 6:**
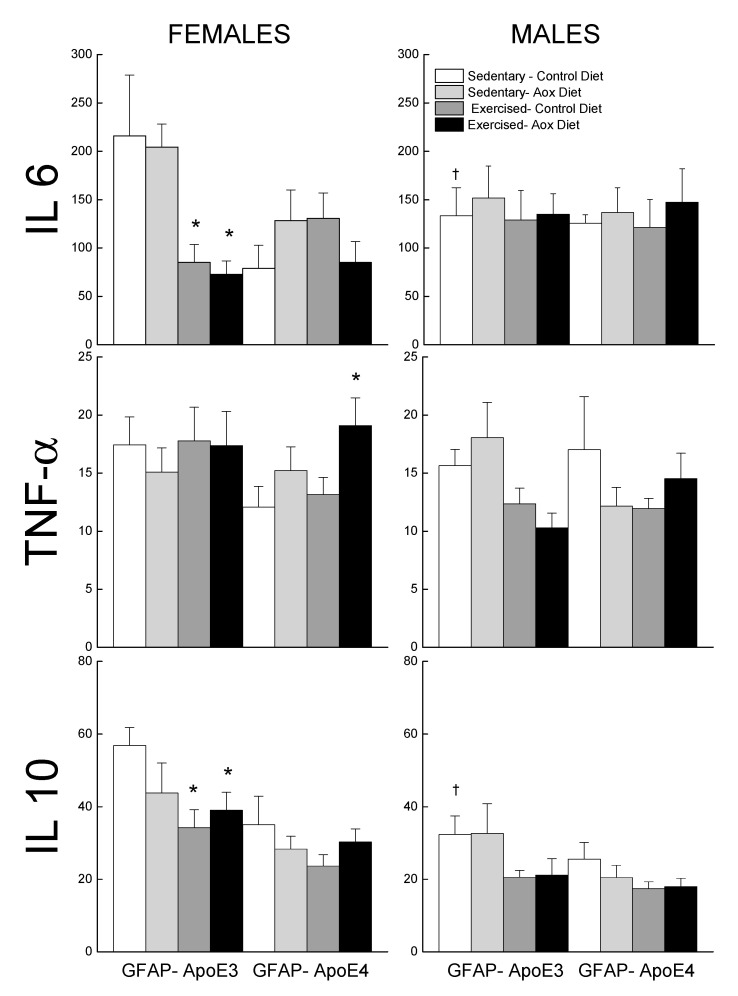
Effect of exercise and antioxidants on markers of inflammation in male and female GFAP-ApoE3 and GFAP-ApoE4 mice. Each value represents mean ± SEM, *n* = 5–9. * *p* < 0.05 vs. sex- and strain-matched Sed-Con groups; † *p* < 0.05 comparing strain-matched Sed-Con males and females.

Levels of IL6, TNFα, and IL10 were measured in the plasma and are reported in Figure 6. For IL6, the effects of treatments were seen in the GFAP-ApoE3 females, with a sex by treatment interaction approaching significance (*p* = 0.055). The female groups that exercised had lower levels of IL6 than the control groups. There was no effect of sex, strain, or treatment on TNFα levels (all *p* > 0.09), though the Ex-Aox GFAP-ApoE4 females had higher levels than the controls (*p* < 0.05).

## 4. Discussion

The main findings of this study were: (1) there were strain differences for most motor functions, but no major differences for strength, balance, or cognition; (2) there were strain and test-dependent differences in response to the treatments: GFAP-ApoE3 mice were responsive on bridge and active avoidance, while GFAP-ApoE3 mice were on spatial learning and memory; (4) the most effective treatment was exercise, and no major additive or antagonistic effects were observed with antioxidant intake.

The current study provided a comprehensive phenotype of this mouse model, and its response to non-conventional therapeutic interventions. Anxiety, the most common non-cognitive symptom of AD [32] and associated with impaired daily activities [33], is often managed with benzodiazepines, which can lead to further cognitive and motor function declines [34,35]. Therefore, identifying non-pharmacotherapeutic agents to reduce anxiety could be an overall positive approach towards managing anxiety symptoms in AD without the added risk of furthering the functional declines. Previously, we determined that at 4 months of age, GFAP-ApoE4 mice were less anxious than GFAP-ApoE3 mice [30]; however, in the current study of 14-month-old mice, that difference subsided. This may indicate a pleiotropic effect of the ApoE genotype on anxiety levels, as has been described with cognitive function [30,36]. These data contrast with a previous study reporting higher anxiety among adult GFAP-ApoE4 mice [37]. Neither antioxidant nor exercise treatment affected the anxiety levels of the mice. In young mice, antioxidants increased the anxiety of the GFAP-ApoE4 mice, suggesting an age-dependent response to interventions [30]. Overall, the GFAP-ApoE3 mice were more active than the GFAP-ApoE4 mice, which is consistent with a previous report [38], and the treatments had no to minimal effects. Other complementary and alternative medicines have also been studied as non-traditional therapies alone or in combination with exercise. More specifically, in a pilot study, depression in AD patients was reduced when they combined Shiatsu with physical activity for 10 months [39].

During its early phase, AD is often associated with motor function impairments. Pathological changes in the motor cortex, striatum, cerebellum, or substantia nigra might be responsible for motor decline in AD [40,41]. The presence of ApoE4 doubles the rate of motor decline associated with aging [7]. Motor declines were observed in our mouse model with GFAP-ApoE4 mice exhibiting decreased activity, reflexes, and coordination. Similar effects on coordinated running were reported, in which the *APOE*4 mice performed poorly [42]. Repetitive transcranial magnetic stimulation suggested that ApoE4 is a critical determinant of the response to conditioning insults, especially for motor and cognitive brain networks [43]. Exercise and/or antioxidants improved the motor learning of the GFAP-ApoE3 males but not of the females or GFAP-ApoE4 mice. At younger ages, exercise training was associated with a reversal of the ApoE4-associated deficits in coordinated running [31]. The lack of response to treatment at an older age again suggests an age-dependent response to treatment. Interestingly, treatments improved the balance of the GFAP-ApoE3 mice, but not of the GFAP-ApoE4 mice. This strain-dependent effect was previously observed in younger mice [31], though the effects were not as large, most likely due to better performance of the young mice. Furthermore, exercise was more successful at improving motor function than antioxidants alone, indicative of a more promising therapeutic. Combining exercise and antioxidants did not lead to additive or antagonistic effects, as seen at younger ages on motor function [31].

Different mouse models expressing human ApoE4 exhibited poor spatial learning and memory [36,38,42]. Our data also suggest a significant decline in spatial learning and memory in GFAP-ApoE4 mice compared to GFAP-ApoE3 mice, a difference that was not observed in younger mice [30]. Interestingly, the majority of that effect comes from the males, as females did not show any differences. This may reflect sex differences in performance or that perhaps a water maze is not useful to detect cognitive differences in females. The treatments did not improve the performance of the GFAP-ApoE3 mice but did improve that of the GFAP-ApoE4 mice. Exercise improved more aspects of water maze performance than antioxidants alone, again supporting the fact that exercise is a better therapy. Improvements in cognition with exercise associated with the ApoE4 genotype have been shown previously [44]. Recent studies have implicated a potential cross-talk between the brain and muscles, with exercise-induced mediators being released, leading to the neuroprotection and brain function improvements associated with exercise. FNDC5/irisin is a myokine released upon exercising that has been associated with improvements in synaptic activity and memory in the APP/PS1 AD mouse model [45]. While our exercise paradigm differed from that study, it would be of interest to determine in future studies using our training paradigm if the FNDC5/irisin pathway is also activated in our regimen.

AD symptoms start with the loss of non-spatial short-term/working memory [46]. While we found strain differences in the spatial task, both GFAP-ApoE3 and ApoE4 mice performed similarly in the non-spatial task used. This is in contrast to previous studies that have identified genotype differences in working memory, which deteriorated in an ε4 allele dose-dependent manner in humans [47]. The GFAP-ApoE3 mice were more responsive to the treatments than the GFAP-ApoE4 mice, and additive effects were observed in the reversal phase for males. Other studies have also determined that exercise can improve short-term/working memory task performance [48,49]. These treatment effects were also observed previously with young GFAP-ApoE3 mice [30], however, while the treatments improved the performance of the young GFAP-ApoE4 mice [30], they did not have any effect on the older mice in the current study. These outcomes on cognitive function suggest that the type of memory, along with the age and genotype, can affect the outcome of interventions.

Contrary to expectation, catalase activity was reduced in brain regions in mice that were exercised, especially the ones combined with antioxidants. Reports on the effects of exercise on catalase activity in the brain are conflicting: from no effect [50] to up-regulation as a result of the oxidative bursts associated with acute exercise [51]. Catalase may be activated upon acute but not chronic exercise. Furthermore, supplementation with antioxidants may have led to a sparing of catalase or a feedback down-regulation of catalase. Plasma-based inflammatory biomarkers are often suggested to be future biomarkers for AD progression and treatment response. The effects of exercise on inflammation are ambiguous, and depend on the focus of inflammation (peripheral (plasma) vs. central (brain)). Studies focused on long-term exercise observed higher IL6 in the brain [52] compared to plasma, while no such change was noticed in TNFα. Some studies demonstrated lower IL6 in the brain after prolonged exercise without affecting plasma IL6 levels [53]. IL6 was positively related to IL10 in the exercise training context [54]. We also observed similar a relation of lowered IL6 in the presence of chronic exercise and associated lowered IL10 levels in GFAP-ApoE3 female mice. On the contrary, treadmill training previously reported higher IL10 and lower TNFα in plasma [55]. Differences between the sexes may be due to hormone influences, as females see a decrease in circulating hormones, which can have repercussion on the inflammatory response [56], and/or may be due to differences in oxidative stress levels and homeostasis [57].

The presence of ApoE4 is associated with an increased risk of developing late-onset AD, however, clinically, it does not seem to always be true with AD patients not carrying the ε4 and vice versa, suggesting a more complicated picture and gene involvement in AD etiology [58]. Furthermore, the presence of one ε4 allele may be insufficient to increase the risk in individuals 65 and younger [59]. Lastly, ApoE4 and other mutations of the known AD genes, such as APP, PSEN1, and PSEN2, only account for 5% of early-onset AD cases [58]. Therefore, the outcomes of this study would need to be further studied in other models of AD, especially relating to early-onset dementia in order to generalize them to all AD patients.

While it remains under debate that these non-pharmacological therapies are effective in reducing neurodegeneration, it is possible that they affect the vascular contribution to these diseases. By managing and controlling the vascular risk factor via a healthy lifestyle, such as exercising and improving one’s diet, it is likely to reduce vascular cognitive impairments and reduce the advance to neurodegenerative diseases and dementias [60]. These non-pharmacological therapies may be working via the control and management of vascular risk, leading to improved cognition and diminished neurodegenerative disorders, such as AD.

Our study has several limitations: (1) the model used may not be generalizable to all AD patients, as it focused on the ApoE genotype and on a glial model of ApoE expression; (2) the limited oxidative stress measurements, as such glutathione levels or protein damage measurements would have strengthened the role of oxidative stress in the study [61]; (3) the involvement of other pathways, such as the irisin-dependent pathway involving muscle–brain cross-talks, were not studied and may be of importance to determine the mechanism of action of our exercise paradigm; (4) a dose–response component of our exercise paradigm would be of interest to determine the translational impact of our training regimen [62].

## 5. Conclusions

Exercise was the most consistent treatment effective at improving motor and cognitive function, and the addition of antioxidants did not lead to major additive or antagonistic effects. ApoE4 mice were less responsive to the treatments than the ApoE3 mice, suggesting a genotype-dependent response to interventions. Therefore, factors such as sex, age, genotype, and chosen tests need to be carefully incorporated into preclinical studies of interventions to improve brain function during aging or neurodegenerative diseases.

## Supplementary Materials

The following are available online at https://www.mdpi.com/2076-3921/9/6/553/s1, Figure S1: Effect of exercise and antioxidant on performance on the elevated plus maze in male and female GFAP-ApoE3 and GFAP-ApoE4 mice, Figure S2: Effect of exercise and antioxidant on spontaneous activity in male and female GFAP-ApoE3 and GFAP-ApoE4 mice, Figure S3: Effect of exercise and antioxidant on reflexes in male and female GFAP-ApoE3 and GFAP-ApoE4 mice, Figure S4: Effect of exercise and antioxidant on catalase activity from different brain regions from GFAP-ApoE3 and GFAP-ApoE4 mice.

## References

[1] Riedel B.C., Thompson P.M., Brinton R.D. (2016). Age, APOE and sex: Triad of risk of Alzheimer’s disease. J. Steroid. Biochem. Mol. Biol..

[2] Yin J.X., Turner G.H., Lin H.J., Coons S.W., Shi J. (2011). Deficits in spatial learning and memory is associated with hippocampal volume loss in aged apolipoprotein E4 mice. J. Alzheimers Dis. JAD.

[3] Hartman R., Wozniak D., Nardi A., Olney J., Sartorius L., Holtzman D. (2001). Behavioral Phenotyping of GFAP-ApoE3 and -ApoE4 Transgenic Mice: ApoE4 Mice Show Profound Working Memory Impairments in the Absence of Alzheimer’s-like Neuropathology. Exp. Neurol..

[4] Johnson L.A., Olsen R.H., Merkens L.S., DeBarber A., Steiner R.D., Sullivan P.M., Maeda N., Raber J. (2014). Apolipoprotein E-low density lipoprotein receptor interaction affects spatial memory retention and brain ApoE levels in an isoform-dependent manner. Neurobiol. Dis..

[5] Buchman A.S., Bennett D.A. (2011). Loss of motor function in preclinical Alzheimer’s disease. Expert Rev. Neurother..

[6] Hebert L.E., Bienias J.L., McCann J.J., Scherr P.A., Wilson R.S., Evans D.A. (2010). Upper and lower extremity motor performance and functional impairment in Alzheimer’s disease. Am. J. Alzheimers Dis. Other Demen..

[7] Buchman A.S., Boyle P.A., Wilson R.S., Beck T.L., Kelly J.F., Bennett D.A. (2009). Apolipoprotein E e4 allele is associated with more rapid motor decline in older persons. Alzheimer Dis. Assoc. Disord..

[8] Luca M., Luca A. (2019). Oxidative Stress-Related Endothelial Damage in Vascular Depression and Vascular Cognitive Impairment: Beneficial Effects of Aerobic Physical Exercise. Oxidative Med. Cell. Longev..

[9] Pratico D., Sung S. (2004). Lipid peroxidation and oxidative imbalance: Early functional events in Alzheimer’s disease. J. Alzheimers Dis. JAD.

[10] Zito G., Polimanti R., Panetta V., Ventriglia M., Salustri C., Siotto M.C., Moffa F., Altamura C., Vernieri F., Lupoi D. (2013). Antioxidant status and APOE genotype as susceptibility factors for neurodegeneration in Alzheimer’s disease and vascular dementia. Rejuvenation Res..

[11] Yasuno F., Tanimukai S., Sasaki M., Ikejima C., Yamashita F., Kodama C., Mizukami K., Asada T. (2012). Combination of antioxidant supplements improved cognitive function in the elderly. J. Alzheimers Dis. JAD.

[12] Roitto H.M., Kautiainen H., Ohman H., Savikko N., Strandberg T.E., Raivio M., Laakkonen M.L., Pitkala K.H. (2018). Relationship of Neuropsychiatric Symptoms with Falls in Alzheimer’s Disease—Does Exercise Modify the Risk?. J. Am. Geriatr. Soc..

[13] Lin T.W., Tsai S.F., Kuo Y.M. (2018). Physical Exercise Enhances Neuroplasticity and Delays Alzheimer’s Disease. Brain Plast..

[14] Cui M.Y., Lin Y., Sheng J.Y., Zhang X., Cui R.J. (2018). Exercise Intervention Associated with Cognitive Improvement in Alzheimer’s Disease. Neural. Plast..

[15] Head D., Bugg J.M., Goate A.M., Fagan A.M., Mintun M.A., Benzinger T., Holtzman D.M., Morris J.C. (2012). Exercise Engagement as a Moderator of the Effects of APOE Genotype on Amyloid Deposition. Arch. Neurol..

[16] Brown B.M., Peiffer J.J., Martins R.N. (2013). Multiple effects of physical activity on molecular and cognitive signs of brain aging: Can exercise slow neurodegeneration and delay Alzheimer’s disease?. Mol. Psychiatry.

[17] Bouzid M.A., Hammouda O., Matran R., Robin S., Fabre C. (2014). Low intensity aerobic exercise and oxidative stress markers in older adults. J. Aging Phys. Act..

[18] Marosi K., Bori Z., Hart N., Sarga L., Koltai E., Radak Z., Nyakas C. (2012). Long-term exercise treatment reduces oxidative stress in the hippocampus of aging rats. Neuroscience.

[19] Verrusio W., Andreozzi P., Marigliano B., Renzi A., Gianturco V., Pecci M.T., Ettorre E., Cacciafesta M., Gueli N. (2014). Exercise training and music therapy in elderly with depressive syndrome: A pilot study. Complement. Med..

[20] Marais L., Stein D.J., Daniels W.M. (2009). Exercise increases BDNF levels in the striatum and decreases depressive-like behavior in chronically stressed rats. Metab. Brain Dis..

[21] Schwenk M., Dutzi I., Englert S., Micol W., Najafi B., Mohler J., Hauer K. (2014). An intensive exercise program improves motor performances in patients with dementia: Translational model of geriatric rehabilitation. J. Alzheimers Dis. JAD.

[22] Zieschang T., Schwenk M., Oster P., Hauer K. (2013). Sustainability of motor training effects in older people with dementia. J. Alzheimers Dis. JAD.

[23] Hauer K., Hildebrandt W., Sehl Y., Edler L., Oster P., Droge W. (2003). Improvement in muscular performance and decrease in tumor necrosis factor level in old age after antioxidant treatment. J. Mol. Med..

[24] Faber M.J., Bosscher R.J., Chin A.P.M.J., van Wieringen P.C. (2006). Effects of exercise programs on falls and mobility in frail and pre-frail older adults: A multicenter randomized controlled trial. Arch. Phys. Med. Rehabil..

[25] Siciliano G., Chico L., Lo Gerfo A., Simoncini C., Schirinzi E., Ricci G. (2020). Exercise-Related Oxidative Stress as Mechanism to Fight Physical Dysfunction in Neuromuscular Disorders. Front. Physiol..

[26] Cetin E., Top E.C., Sahin G., Ozkaya Y.G., Aydin H., Toraman F. (2010). Effect of vitamin E supplementation with exercise on cognitive functions and total antioxidant capacity in older people. J. Nutr. Health Aging.

[27] Jolitha A.B., Subramanyam M.V., Asha Devi S. (2006). Modification by vitamin E and exercise of oxidative stress in regions of aging rat brain: Studies on superoxide dismutase isoenzymes and protein oxidation status. Exp. Gerontol..

[28] Wu A., Ying Z., Gomez-Pinilla F. (2008). Docosahexaenoic acid dietary supplementation enhances the effects of exercise on synaptic plasticity and cognition. Neuroscience.

[29] Ristow M., Zarse K., Oberbach A., Kloting N., Birringer M., Kiehntopf M., Stumvoll M., Kahn C.R., Bluher M. (2009). Antioxidants prevent health-promoting effects of physical exercise in humans. Proc. Natl. Acad. Sci. USA.

[30] Chaudhari K., Wong J.M., Vann P.H., Sumien N. (2014). Exercise training and antioxidant supplementation independently improve cognitive function in adult male and female GFAP-*APOE* mice. J. Sport Health Sci..

[31] Chaudhari K., Wong J.M., Vann P.H., Sumien N. (2016). Exercise, but not antioxidants, reversed ApoE4-associated motor impairments in adult GFAP-ApoE mice. Behav. Brain Res..

[32] Ferretti L., McCurry S.M., Logsdon R., Gibbons L., Teri L. (2001). Anxiety and Alzheimer’s disease. J. Geriatr. Psychiatry Neurol..

[33] Teri L., Ferretti L.E., Gibbons L.E., Logsdon R.G., McCurry S.M., Kukull W.A., McCormick W.C., Bowen J.D., Larson E.B. (1999). Anxiety of Alzheimer’s disease: Prevalence, and comorbidity. J. Gerontol. A Biol. Sci. Med. Sci..

[34] Hetland A., Carr D.B. (2014). Medications and impaired driving. Ann. Pharmacother..

[35] Stewart S.A. (2005). The effects of benzodiazepines on cognition. J. Clin. Psychiatry.

[36] Raber J., Wong D., Buttini M., Orth M., Bellosta S., Pitas R.E., Mahley R.W., Mucke L. (1998). Isoform-specific effects of human apolipoprotein E on brain function revealed in ApoE knockout mice: Increased susceptibility of females. Proc. Natl. Acad. Sci. USA.

[37] Raber J. (2007). Role of apolipoprotein E in anxiety. Neural. Plast..

[38] Bour A., Grootendorst J., Vogel E., Kelche C., Dodart J.C., Bales K., Moreau P.H., Sullivan P.M., Mathis C. (2008). Middle-aged human apoE4 targeted-replacement mice show retention deficits on a wide range of spatial memory tasks. Behav. Brain Res..

[39] Lanza G., Centonze S.S., Destro G., Vella V., Bellomo M., Pennisi M., Bella R., Ciavardelli D. (2018). Shiatsu as an adjuvant therapy for depression in patients with Alzheimer’s disease: A pilot study. Complement. Med..

[40] Schneider J.A., Li J.L., Li Y., Wilson R.S., Kordower J.H., Bennett D.A. (2006). Substantia nigra tangles are related to gait impairment in older persons. Ann. Neurol..

[41] Mavroudis I.A., Fotiou D.F., Adipepe L.F., Manani M.G., Njau S.D., Psaroulis D., Costa V.G., Baloyannis S.J. (2010). Morphological changes of the human purkinje cells and deposition of neuritic plaques and neurofibrillary tangles on the cerebellar cortex of Alzheimer’s disease. Am. J. Alzheimers Dis. Other Demen..

[42] van Meer P., Acevedo S., Raber J. (2007). Impairments in spatial memory retention of GFAP-apoE4 female mice. Behav. Brain Res..

[43] Mkopi A., Range N., Lwilla F., Egwaga S., Schulze A., Geubbels E., van Leth F. (2012). Adherence to tuberculosis therapy among patients receiving home-based directly observed treatment: Evidence from the United Republic of Tanzania. PLoS ONE.

[44] Nichol K., Deeny S.P., Seif J., Camaclang K., Cotman C.W. (2009). Exercise improves cognition and hippocampal plasticity in APOE epsilon4 mice. Alzheimers Dement. J. Alzheimers Assoc..

[45] Lourenco M.V., Frozza R.L., de Freitas G.B., Zhang H., Kincheski G.C., Ribeiro F.C., Goncalves R.A., Clarke J.R., Beckman D., Staniszewski A. (2019). Exercise-linked FNDC5/irisin rescues synaptic plasticity and memory defects in Alzheimer’s models. Nat. Med..

[46] Stopford C.L., Thompson J.C., Neary D., Richardson A.M., Snowden J.S. (2012). Working memory, attention, and executive function in Alzheimer’s disease and frontotemporal dementia. Cortex.

[47] Greenwood P.M., Lambert C., Sunderland T., Parasuraman R. (2005). Effects of apolipoprotein E genotype on spatial attention, working memory, and their interaction in healthy, middle-aged adults: Results From the National Institute of Mental Health’s BIOCARD study. Neuropsychology.

[48] Kim S.E., Ko I.G., Shin M.S., Kim C.J., Jin B.K., Hong H.P., Jee Y.S. (2013). Treadmill exercise and wheel exercise enhance expressions of neutrophic factors in the hippocampus of lipopolysaccharide-injected rats. Neurosci. Lett..

[49] Deeny S.P., Poeppel D., Zimmerman J.B., Roth S.M., Brandauer J., Witkowski S., Hearn J.W., Ludlow A.T., Contreras-Vidal J.L., Brandt J. (2008). Exercise, APOE, and working memory: MEG and behavioral evidence for benefit of exercise in epsilon4 carriers. Biol. Psychol..

[50] Radak Z., Kumagai S., Taylor A.W., Naito H., Goto S. (2007). Effects of exercise on brain function: Role of free radicals. Appl. Physiol. Nutr. Metab. Physiol. Appl. Nutr. Metab..

[51] Bogdanis G.C., Stavrinou P., Fatouros I.G., Philippou A., Chatzinikolaou A., Draganidis D., Ermidis G., Maridaki M. (2013). Short-term high-intensity interval exercise training attenuates oxidative stress responses and improves antioxidant status in healthy humans. Food Chem. Toxicol. Int. J. Publ. Br. Ind. Biol. Res. Assoc..

[52] Nybo L., Nielsen B., Pedersen B.K., Moller K., Secher N.H. (2002). Interleukin-6 release from the human brain during prolonged exercise. J. Physiol..

[53] Chennaoui M., Drogou C., Gomez-Merino D. (2008). Effects of physical training on IL-1beta, IL-6 and IL-1ra concentrations in various brain areas of the rat. Eur. Cytokine Netw..

[54] Pedersen B.K. (2006). The anti-inflammatory effect of exercise: Its role in diabetes and cardiovascular disease control. Essays Biochem..

[55] Goldhammer E., Tanchilevitch A., Maor I., Beniamini Y., Rosenschein U., Sagiv M. (2005). Exercise training modulates cytokines activity in coronary heart disease patients. Int. J. Cardiol..

[56] Villa A., Vegeto E., Poletti A., Maggi A. (2016). Estrogens, Neuroinflammation, and Neurodegeneration. Endocr. Rev..

[57] Torrens-Mas M., Pons D.G., Sastre-Serra J., Oliver J., Roca P. (2020). Sexual hormones regulate the redox status and mitochondrial function in the brain. Pathological implications. Redox Biol..

[58] D’Argenio V., Sarnataro D. (2020). New Insights into the Molecular Bases of Familial Alzheimer’s Disease. J. Pers. Med..

[59] Cacace R., Sleegers K., Van Broeckhoven C. (2016). Molecular genetics of early-onset Alzheimer’s disease revisited. Alzheimers Dement. J. Alzheimers Assoc..

[60] Ngandu T., Lehtisalo J., Solomon A., Levalahti E., Ahtiluoto S., Antikainen R., Backman L., Hanninen T., Jula A., Laatikainen T. (2015). A 2 year multidomain intervention of diet, exercise, cognitive training, and vascular risk monitoring versus control to prevent cognitive decline in at-risk elderly people (FINGER): A randomised controlled trial. Lancet.

[61] Fisher-Wellman K., Bloomer R. (2009). Acute exercise and oxidative stress: A 30 year history. Dyn. Med..

[62] Gronwald T., de Bem Alves A.C., Murillo-Rodriguez E., Latini A., Schuette J., Budde H. (2019). Standardization of exercise intensity and consideration of a dose-response is essential. Commentary on “Exercise-linked FNDC5/irisin rescues synaptic plasticity and memory defects in Alzheimer’s models”, by Lourenco et al., published 2019 in Nature Medicine. J. Sport Health Sci..

