# Supplementary material for: ApoE Genotype-Dependent Response to Antioxidant and Exercise Interventions on Brain Function
Source: Antioxidants (Basel). 2020 Jun 25;9(6):553. doi: 10.3390/antiox9060553 (PMC7346214; doi:10.3390/antiox9060553)
Supplement: Supplementary file 1 [file antioxidants-09-00553-s001.pdf]

antioxidants

MDPI

Supplementary Figures

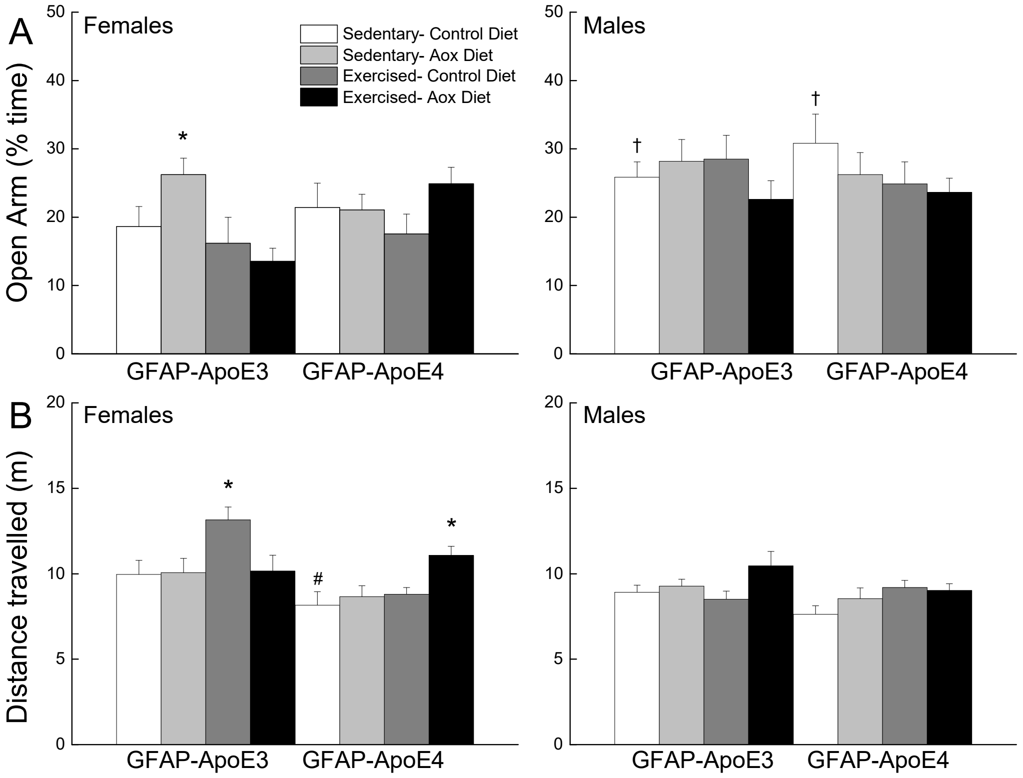

Figure S1. Effect of exercise and antioxidant on performance on the elevated plus maze in male and female GFAP-ApoE3 and GFAP-ApoE4 mice. Each value represents mean ± SEM, $n = 8–16$. * $p < 0.05$ vs. sex- and strain-matched Sed-Con groups; # $p < 0.05$ comparing sex-matched Sed-Con GFAP-ApoE3 and GFAP-ApoE4; † $p < 0.05$ comparing strain-matched Sed-Con males and females.

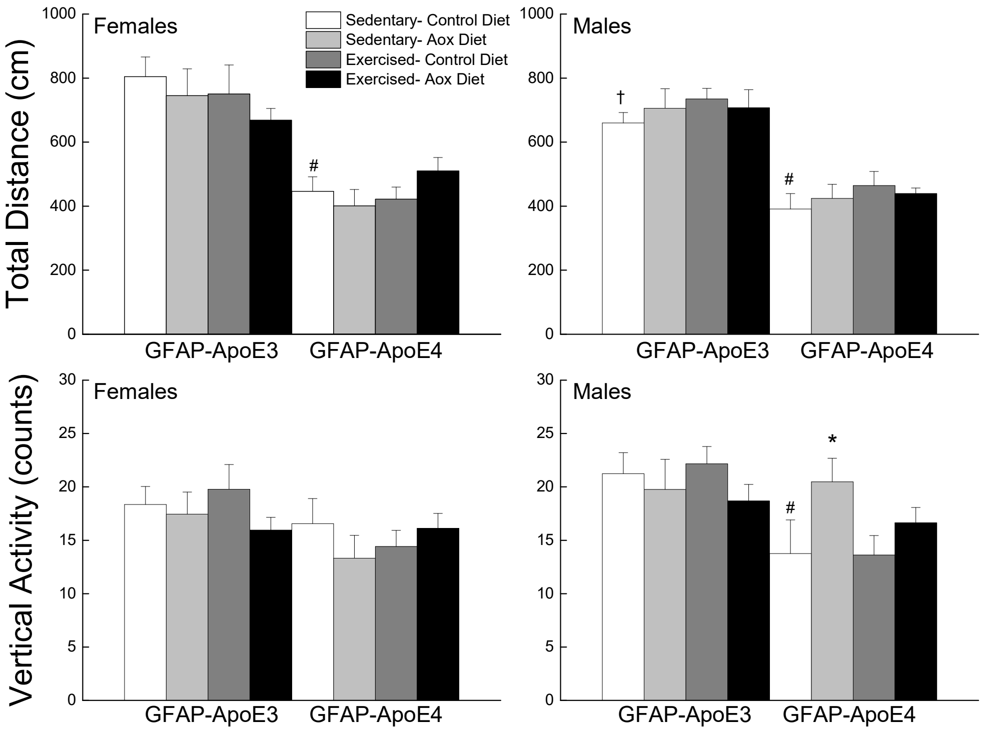

Figure S2. Effect of exercise and antioxidant on spontaneous activity in male and female GFAP-ApoE3 and GFAP-ApoE4 mice. Each value represents mean ± SEM, $n = 8–16$. * $p < 0.05$ vs. sex- and strain-matched Sed-Con groups; # $p < 0.05$ comparing sex-matched Sed-Con GFAP-ApoE3 and GFAP-ApoE4.

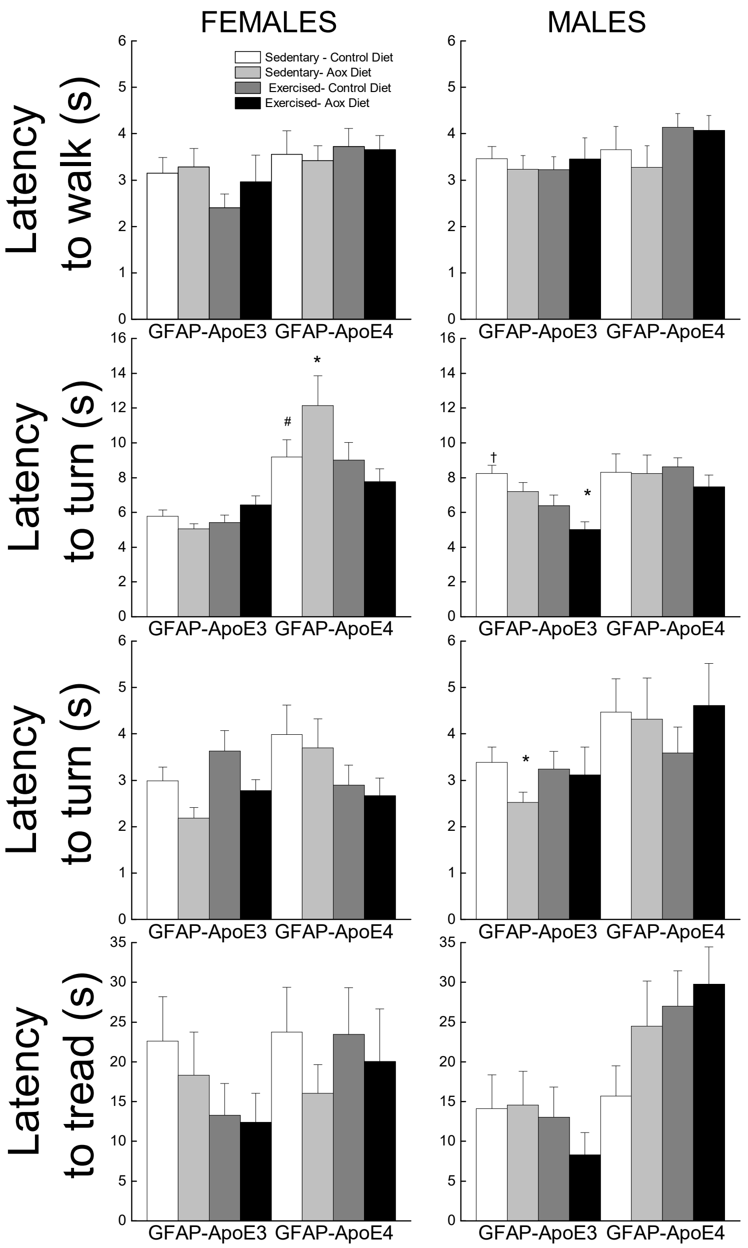

Figure S3. Effect of exercise and antioxidant on reflexes in male and female GFAP-ApoE3 and GFAP-ApoE4 mice. Each value represents mean ± SEM, n = 8–16. * $p < 0.05$ vs. sex- and strain-matched Sed-Con groups; # $p < 0.05$ comparing sex-matched Sed-Con GFAP-ApoE3 and GFAP-ApoE4; † $p < 0.05$ comparing strain-matched Sed-Con males and females.

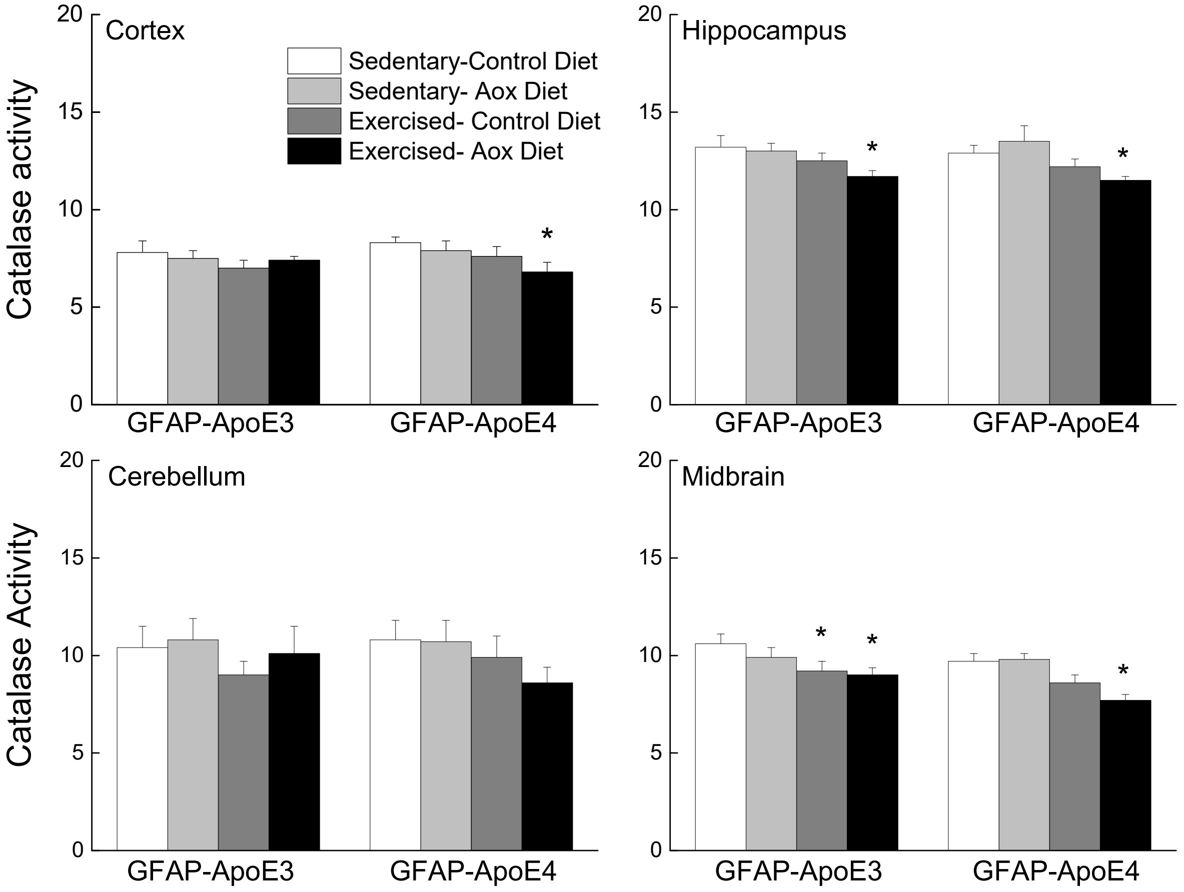

Figure S4. Effect of exercise and antioxidant on catalase activity from different brain regions from GFAP-ApoE3 and GFAP-ApoE4 mice. Each value represents mean ± SEM, n = 8–16. * $p < 0.05$ vs. sex- and strain-matched Sed-Con groups.
